# Effects of host‐derived chemokines on the motility and viability of *Trypanosoma brucei*

**DOI:** 10.1111/pim.12609

**Published:** 2018-12-27

**Authors:** Omar A. Alfituri, Olumide Ajibola, James M. Brewer, Paul Garside, Robert A. Benson, Tamlyn Peel, Liam J. Morrison, Neil A. Mabbott

**Affiliations:** ^1^ The Roslin Institute and Royal (Dick) School of Veterinary Sciences University of Edinburgh Edinburgh UK; ^2^ Wellcome Centre for Molecular Parasitology Institute of Infection, Immunity and Inflammation College of Medicine and Veterinary Medicine Glasgow UK; ^3^ Department of Microbiology Federal University Birnin Kebbi Birnin Kebbi Nigeria; ^4^ Centre for Inflammation Biology and Cancer Immunology Faculty of Life Sciences King's College London London UK

**Keywords:** chemokines, chemotaxis, cytotoxicity, intravital imaging, skin, lymphatics, *Trypanosoma brucei*

## Abstract

African trypanosomes (*Trypanosoma brucei* spp.) are extracellular, hemoflagellate, protozoan parasites. Mammalian infection begins when the tsetse fly vector injects trypanosomes into the skin during blood feeding. The trypanosomes then reach the draining lymph nodes before disseminating systemically. Intravital imaging of the skin post‐tsetse fly bite revealed that trypanosomes were observed both extravascularly and intravascularly in the lymphatic vessels. Whether host‐derived cues play a role in the attraction of the trypanosomes towards the lymphatic vessels to aid their dissemination from the site of infection is not known. Since chemokines can mediate the attraction of leucocytes towards the lymphatics, in vitro chemotaxis assays were used to determine whether chemokines might also act as chemoattractants for trypanosomes. Although microarray data suggested that the chemokines CCL8, CCL19, CCL21, CCL27 and CXCL12 were highly expressed in mouse skin, they did not stimulate the chemotaxis of *T brucei*. Certain chemokines also possess potent antimicrobial properties. However, none of the chemokines tested exerted any parasiticidal effects on *T brucei*. Thus, our data suggest that host‐derived chemokines do not act as chemoattractants for *T brucei*. Identification of the mechanisms used by trypanosomes to establish host infection will aid the development of novel approaches to block disease transmission.

## 1 INTRODUCTION

African trypanosomes are single‐cell extracellular hemoflagellate protozoan parasites and are transmitted between mammalian hosts via blood‐feeding tsetse flies of the genus *Glossina*. The *Trypanosoma brucei rhodesiense* and *T b gambiense* subspecies cause human African trypanosomiasis in endemic regions within the tsetse fly belt across sub‐Saharan Africa. Animal African trypanosomiasis is caused by *Trypanosoma congolense*,* Trypanosoma vivax* and *T brucei* and inflicts substantial economic strains on the African livestock industry.

The parasitic life cycle within the mammalian host is initiated by the intradermal injection of metacyclic trypomastigotes by the tsetse fly vector. The extracellular parasites then reach the draining lymph nodes, presumably via invasion of the afferent lymphatics and then disseminate systemically.^1^, ^2^ During this process, the parasites also undergo morphological change into long slender bloodstream forms that are adapted for survival within the mammalian host. Early studies showed that in both cattle and goats infected with *T vivax* by tsetse fly bite, the parasites were detectable in the draining pre‐scapular lymph nodes earlier than in peripheral blood during the initial period of infection.^3^, ^4^ This progression has also been replicated in mice.^2^ Following their intradermal injection by tsetse fly bite, *T brucei* parasites were similarly first detected within the draining lymph nodes by 18 hours, and subsequently detected in the blood by 42 hours.^2^ These data raise the hypothesis that intradermally injected trypanosomes initially infect the local lymphatics within the skin before subsequently infecting the bloodstream and spreading systemically. The currently licensed drugs that are available to treat trypanosomiasis have dangerous side‐effects, and drug‐resistance is an increasing problem. A fuller understanding of the mechanisms used by African trypanosomes to enable their systemic dissemination after infection would aid the development of novel approaches to block disease pathogenesis and transmission.

In the current study, intravital imaging revealed the novel finding that after injection into the skin African trypanosomes could be observed both extravascularly and intravascularly within the lymphatic vessels. We therefore tested the hypothesis that host‐derived cues such as chemokines may play a role in the attraction of the trypanosomes towards the lymphatic vessels to enable their dissemination from the site of infection. Chemokines comprise a superfamily of 8‐12 kDa globular proteins that play important roles in the attraction of lymphocytes and leucocytes towards the lymphatics and lymphoid tissues and coordinate their positioning within them.^5^, ^6^ Chemokines mediate their activities through interactions with specific G protein‐coupled chemokine receptors, which trigger intracellular pathways involved in cell motility and activation.^7^ For example, expression of the chemokines CCL19 and CCL21 by local lymphatic endothelial cells mediates the homing of chemokine receptor CCR7 expressing dendritic cells.^8^ In this study, publicly available microarray data sets were analysed to identify genes encoding chemokines that were highly expressed in mouse skin. In vitro assays were then used to test the hypothesis that these host‐derived chemokines may act as chemoattractants for *T brucei*.

In addition to their role in coordinating the chemotaxis and positioning of cells within tissues, many chemokines also possess potent antimicrobial properties, especially against certain pathogenic bacteria and fungi.^9^, ^10^, ^11^, ^12^, ^13^ These antimicrobial chemokines mediate their antimicrobial activities predominantly through the disruption and lysis of the pathogen cell membrane.^9^ Truncated variants of some CXC chemokines have also been reported to be directly bactericidal for *Bacillus subtilis*,* Escherichia coli*,* Lactococcus lactis* and *Staphylococcus aureus*, and fungicidal against *Cryptococcus neoformans*.^14^ The chemokine CCL28 has also been shown to have direct antimicrobial activity against bacteria and fungi, as well as direct parasiticidal effects against the protozoan parasite *Leishmania mexicana,* 15 a kinetoplastid protozoan related to *T brucei*. Therefore, in the current study in vitro assays were also used to determine whether certain chemokines mediated any direct parasiticidal effects against *T brucei*.

## 2 MATERIALS AND METHODS

### 2.1 Trypanosomes

In vitro cultivated monomorphic *T b brucei* Lister 427 strain trypanosomes, pleomorphic *T b brucei* STIB247 strain and *T b brucei* STIB247 trypanosomes^16^ expressing mCherry were used where indicated in this study. Bloodstream‐form Lister 427 parasites were axenically cultivated in vitro at 37°C in the presence of 5% CO_2_ as previously described^17^ using Iscoves modified Dulbecco's medium supplemented with hypoxanthine (1.36 g/mL; Invitrogen), bathocuproinedisulphonic acid disodium salt (2.82 mg/mL; Sigma), thymidine (3.3 mg/mL, Sigma), sodium pyruvate (22 mg/mL, Sigma), L‐cysteine (18.2 mg/mL, Sigma), β‐mercaptoethanol (0.2 mmol/L, Invitrogen), kanamycin (10 mg/mL, Invitrogen), penicillin/streptomycin (100 U/mL, Invitrogen), 10% foetal bovine serum (Invitrogen) and 10% foetal bovine Serum‐Plus (Sigma). Bloodstream‐form STIB247 parasites were cultivated in a modified recipe of the above medium with the addition of glucose (100 mg/mL, Sigma), adenosine (13.4 mg/mL, Sigma), guanosine (14.2 mg/mL, Sigma), methylcellulose (110 mg/mL, Sigma), and 20% foetal bovine serum and 20% foetal bovine Serum‐Plus.

### 2.2 In vivo imaging

All in vivo procedures were carried out at the University of Glasgow in accordance with United Kingdom Home Office regulations under the authority of the appropriate project and personal licenses. Prox‐1 mOrange mice^18^ were anesthetised using a freshly prepared 1:1 mixture of Hypnorm (25 mg/kg) and Hypnovel (12.5 mg/kg) injected intraperitoneally. The hair on the mouse ear to be imaged was removed by applying a hair removal cream (Nair) to the mouse ear for 2 minutes, and then excess was removed with a damp tissue. The mouse was then placed on a custom built imaging platform, and core body temperature was continuously monitored by a rectal probe and maintained by a thermostatically controlled heat mat at 37°C. The mouse ear was immobilised on the imaging platform using glue (3M Vetbond) which was set on the addition of phosphate buffered saline. Prior to imaging, the mouse ear was injected intradermally with 10 μL containing 1 × 10^6^ bloodstream form *T b brucei* STIB247 trypanosomes expressing mCherry.^16^ Multiphoton imaging was performed using a Zeiss LSM7 MP system equipped with both a 10^X^/0.3 NA air and 20^X^/1.0NA water‐immersion objective lens (Zeiss). Two, fully tunable excitation wavelengths were produced by a Titanium/sapphire (Ti/S) solid‐state 2‐photon excitation source (Chameleon Ultra II; Coherent Laser Group), coupled to an optical parametric oscillator (OPO, Coherent Laser Group). A Ti/S laser output of 820 nm and OPO signal of 1030 and 1090 nm provided excitation of mCherry and mOrange. Images were acquired for approximately 15‐20 minutes and 3D tracking was performed using Volocity 6.1.1 (Perkin Elmer) and Imaris 7.6.5 software (Bitplane, Oxford Instruments). Parasite turning angles were calculated using MATLAB software (Mathworks, version R2016b). Images were collected from 5 individual mice and 2‐3 fields of view/mouse.

### 2.3 Microarray data

The NCBI Gene Expression Omnibus database (http://www.ncbi.nlm.nih.gov) was searched for mouse skin expression data sets on the Affymetrix mouse genome 430 2.0 microarray platform. Three independent studies which included normal, uninfected, wild‐type mouse skin were publicly available (GSE17511, GSE7694, GSE27628), and the raw data sets (.cel files) were downloaded. The quality of the raw data was analysed and normalised using Robust Multichip Analysis (RMA EXPRESS; http://rmaexpress.bmbolstad.com/) and annotated using the library file available from Affymetrix (release 36, 13/4/16; http://www.affymetrix.com/).

### 2.4 Chemotaxis assays

All recombinant mouse chemokines used in this study were purchased from Peprotech (London, UK). For chemotaxis assays, triplicate cultures were established as follows: Chemokines were suspended in the relevant trypanosome culture medium and 600 μL added to the corresponding wells of 24‐well plates (Thermo Scientific). Heat‐inactivated chemokines (treatment at 95°C for 5 minutes) or medium alone were used as negative controls. A 3 μm transwell insert (Millipore Europe) was added to each well, and 1 × 10^6^ trypanosomes in 100 μL media subsequently added to the upper chamber. The plates were incubated for 2 hours at 37°C in the presence of 5% CO_2_, and the number of trypanosomes that had passed through the transwell insert pore into the lower chamber was counted in triplicate using an improved Neubauer haemocytometer. Experiments were repeated three times. Mouse splenocytes were also assessed as a positive chemotaxis control. Splenocytes were isolated in fresh RPMI 1640 media (5 mL of penicillin/streptomycin, 5 mL of L‐glutamine and 0.1% fatty acid‐free BSA (Sigma‐Aldrich) in 0.5 L of RPMI solution) by gently mashing them through a 70 μm EASYstrainer cell sieve (Greiner). Next, 1 × 10^5^ splenocytes suspended in 100 μL of media were added to the upper wells of 3 μm pore inserts (Millipore Europe), and 600 μL of chemokine‐containing medium or RPMI 1640 medium was added to the bottom wells. The plates were incubated for 2 hours at 37°C in the presence of 5% CO_2_, and the cells that had passed through the transwell insert pore into the lower chamber collected. The cells were stained for FACS analysis using the following antibodies: anti‐B220/CD45R‐BV605 (RA3‐6B2; Biolegend); anti‐CD3‐PB (145‐2c11; Biolegend); and anti‐CD11c‐PE (N418; BD Pharmingen). Cells were acquired on a BD Fortessa LSR flow cytometer running FACSDiva and analysed using FlowJo 10.1 analysis software (FlowJo LLC).

To determine effects of chemokine treatment on trypanosome motility, *T brucei* Lister 427 parasites were treated with the chemokines CCL21, CCL27 and CCL28 or media alone as above at 37°C in the presence of 5% CO_2_ for 2 hours. The trypanosomes where then transferred to chambered slides (Lab‐Tek Chambered Coverslip no. 1.5; Thermo Scientific), placed on a heated stage, and viewed using a Zeiss Axiovert 100 inverted microscope. Videos of trypanosome motility in each condition (30 secs long, 50 frames/s) were recorded using a Hamamatsu digital camera (Low‐light, Hamamatsu Photonics) and Micro‐manager 18.1.14 imaging software (ImageJ plug‐in, NIH). Trypanosome motility in the videos was then subsequently analysed using Imaris 8.1.2 software (Bitplane, Oxford Instruments). For each treatment condition, data for 90 individual trypanosomes were collected (30 trypanosomes/condition for each of 3 independent experiments).

### 2.5 Cytotoxicity assays

For cytotoxicity assays, triplicate cultures of 8 × 10^5^ trypanosomes in 100 μL medium were added to each well of a 96‐well plate. Chemokine‐containing medium (200 μL/well) was then added to each well at a final concentration of 10, 100 or 500 ng/mL. Heat‐inactivated chemokines (treatment at 95°C for 5 minutes.) or medium alone were used as controls. The plates were incubated for 2 hours at 37°C in the presence of 5% CO_2_, and the number of viable trypanosomes counted in triplicate using an improved Neubauer haemocytometer. Experiments were repeated three times.

### 2.6 Membrane permeability assays

*Trypanosoma brucei* Lister 427 trypanosomes were treated with 500 ng/mL of chemokines or 10 μmol/L of the anti‐trypanosome drug Berenil (diminazine aceturate, as a positive control) in 100 μL of media for 2 hours as described above. The parasites were then diluted 1:2 in PBS containing 4% propidium iodide (PI) and incubated in the dark for 30 minutes on ice. Uptake of PI into cells was then determined using a BD FACS Calibur cytometer and FlowJo 10.1 analysis software.

Trypan blue‐exclusion was used to determine the number of live or dead trypanosomes present following chemokine treatment. Approximately 8 × 10^5^ trypanosomes in 100 μL medium were added to each well of a 96‐well plate. Chemokine‐containing medium (200 μL/well) was then added to each well at a final concentration of 500 ng/mL. The anti‐trypanosome drug Berenil was used as a control at concentrations of 1, 2 and 10 μmol/L. The plates were incubated for 2 hours at 37°C in the presence of 5% CO_2_. Afterwards, the parasites were suspended in trypan blue dye and the number of live and dead trypanosomes counted using a haemocytometer.

### 2.7 Transmission electron microscopy (TEM)

To assess the impact of chemokine treatment on the morphological integrity of the parasites, monomorphic *T brucei* Lister 427 trypanosomes were treated with chemokine‐containing media (500 ng/mL), the trypanocidal drug Berenil (diminazine aceturate, 10 μmol/L) as a positive control or medium alone as a negative control. After 2 hours of exposure, the parasites were then processed for TEM imaging. Samples were washed in PBS to remove excess media and resuspended in 3% gluteraldehyde. Trypanosome pellets were then treated with 0.1 M sodium cacodylate buffer (pH 7.2) for 2 hours and washed three times for 10 minutes in fresh 0.1 M sodium cacodylate buffer. The samples were then post‐fixed in 1% osmium tetroxide in 0.1 M sodium cacodylate for 45 minutes and washed further three times for 10 minutes in fresh 0.1 M sodium cacodylate buffer. The samples were then dehydrated in increasing concentrations of ethanol and subsequently treated with propylene oxide. The samples were then embedded in TAAB 812 resin (TAAB Laboratories Equipment Ltd), and sections (1 μm thick) stained with toluidine blue and viewed under a light microscope. Ultra‐thin sections (60 nm thick) were then cut from the areas of interest and stained in uranyl acetate and lead citrate for being analysed by TEM.

### 2.8 Statistical analysis

All data are derived from three independent experiments. Statistical analyses were performed using GraphPad Prism 6.01 (Graphpad Software, Inc.). Multiple comparisons between multiple groups from independent experiments were analysed using multi‐way ANOVA with Tukey's multiple comparisons test. On each graph, the individual replicates are shown, whereas the horizontal bar represents the mean ± SD.

## 3 RESULTS

### 3.1 Trypanosomes can be found in skin lymphatic vessels after intradermal injection

**Figure 1:**
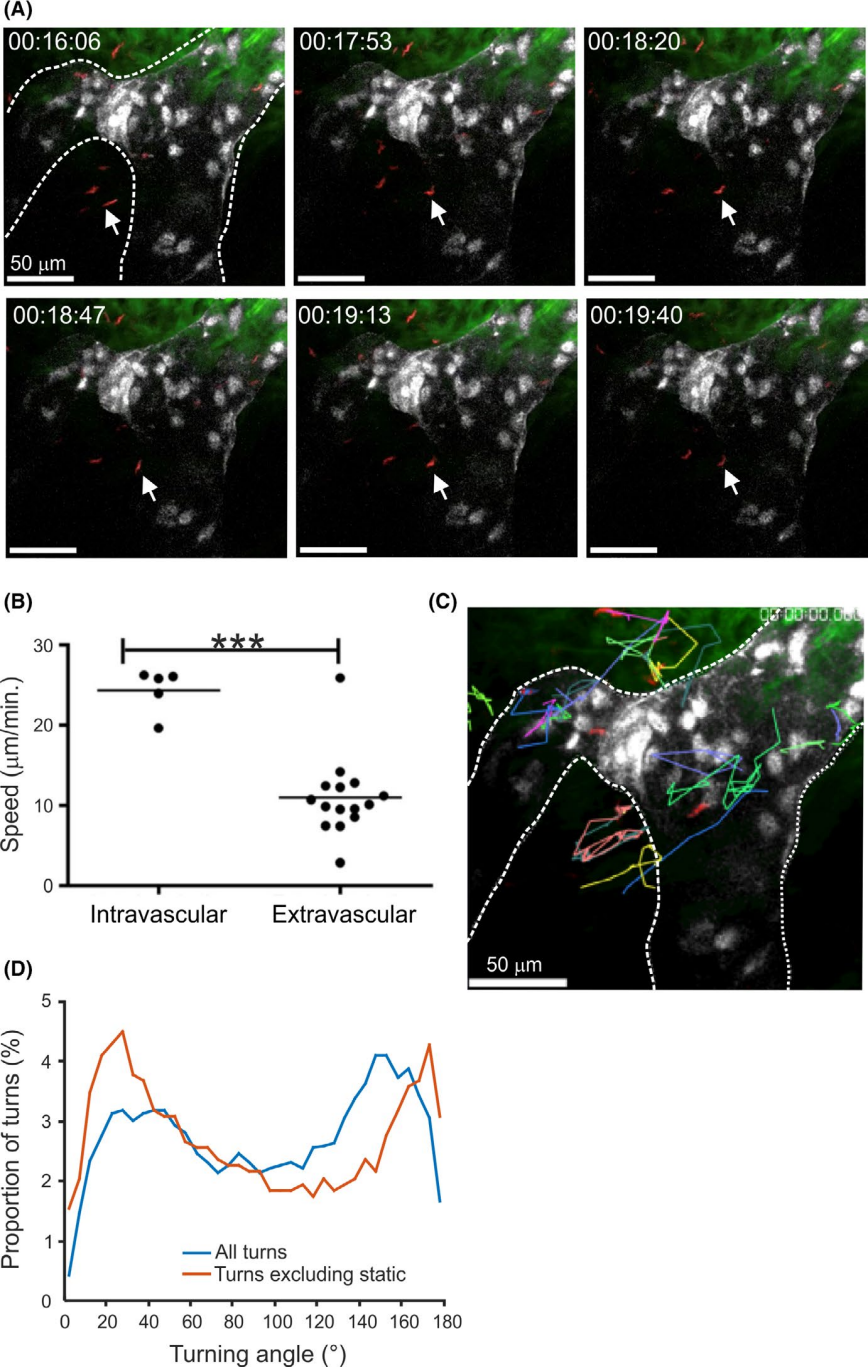
Intravital imaging shows *T brucei* could be observed both extravascularly and intravascularly in the lymphatic vessels after injection into the skin. A, Still images from Movie S1 show *T brucei* parasites (red) present adjacent to and within lymphatic vessels (white) in the skin of Prox1 mOrange reporter mice. B, Analysis of parasite velocities in Movie S1 revealed that intravascular parasites displayed greater speed of movement compared with extravascular parasites. *^**^ *P *<* *0.001. Furthermore, parasites adjacent to lymphatic vessels displayed directional movement towards or away from lymphatic vessels (identified by arrows in (A) and parasite tracking data (C)). D, Analysis of parasite tracks in panel C and Movie S1 confirmed that the parasites had a bimodal distribution of turning angles, suggesting directional movement in the skin. Exclusion of static parasites from the analysis (red line) made this conclusion more evident. Scale bars, 50 μm. White broken lines in A and C outline the lymphatic vessels. Images and movie are representative of 5 mice and 2‐3 fields of view/mouse

A previous study in mice^2^ has shown that following the intradermal injection by tsetse fly bite, *T brucei* parasites were first detected within the draining lymph nodes by 18 hours and subsequently detected in the blood by 42 hours. This raised the hypothesis that trypanosomes initially infect the lymphatic vessels in the skin after intradermal injection to disseminate from the site of infection. We used intravital imaging to visualise trypanosomes following injection into the skin. *T brucei* could be observed both extravascularly and intravascularly in the lymphatic vessels (Figure 1A and Movie S1). The trypanosomes detected within the lymphatics had a significantly higher velocity compared with extra‐lymphatic parasites (Figure**** 1B; *P *<* *0.001). The majority of extravascular parasites appeared to be moving non‐randomly, towards or away from the lymphatic vessels as assessed by turning angle (turning angles 0° or 180°, respectively; (Figure**** 1A, C & D), with individual parasites repeatedly moving towards, then away, then back again. If static parasites were excluded from this analysis (those moving <5 μm between frames), this bimodal distribution was particularly clear (Figure 1D).

### 3.2 Chemokine gene expression in mouse skin

**Figure 2:**
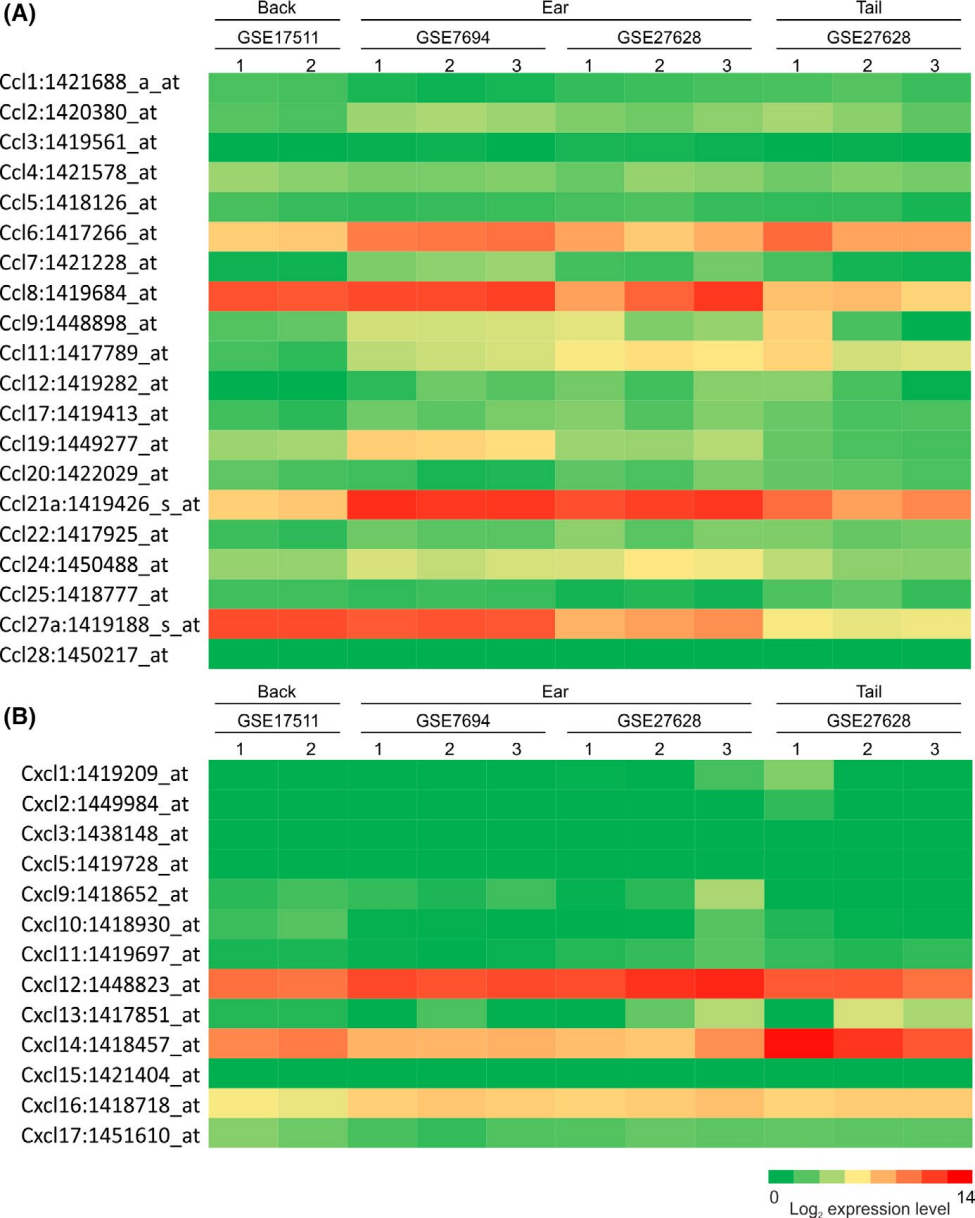
Retrospective comparison of chemokine gene expression in mouse skin. Heat maps show the expression of profile of multiple CCL (A) or CXCL (B) chemokine‐encoding probe sets in the samples of back, ear and tail skin (GEO accession codes: GSE17511; GSE7694; GSE27628). These data were performed on Affymetrix MOE430_2 mouse genome expression arrays (Affymetrix, Santa Clara, CA). Each column represents the mean probe set intensity (log2) for individual data sets (samples) from each source. Representative probe set are shown when multiple probe sets for a gene were present on the arrays

Since trypanosomes were detected within lymphatic vessels after intradermal injection, we hypothesized that the parasites may be responsive to host‐derived cues such as chemokines and use them to aid their dissemination from the site of infection. We therefore compared the expression of chemokine‐encoding genes in mouse skin using publicly available collections of microarray data. Data from three independent studies were analysed (GEO accession codes: GSE17511; GSE7694; GSE27628) comprising a total of 11 individual microarrays (data sets) performed on the Affymetrix mouse genome 430 2.0 platform. This analysis showed that genes encoding the chemokines CCL6, CCL8, CCL21, CCL27, CXCL12, CXCL14 and CXCL16 were expressed highly in the mouse back, ear and tail skin data sets (Figure 2A,B). Of these, the chemokines CCL8, CCL21, CCL27 and CXCL12 were selected for use in subsequent experiments, due to their high expression levels in the mouse skin regions. We also included CCL19 since this chemokine, together with CCL21, contributes to the homing of lymphocytes and leucocytes across the vascular endothelium.^8^, ^19^

### 3.3 Effects of in vitro chemokine exposure on the chemotaxis and motility of *T brucei*

**Figure 3:**
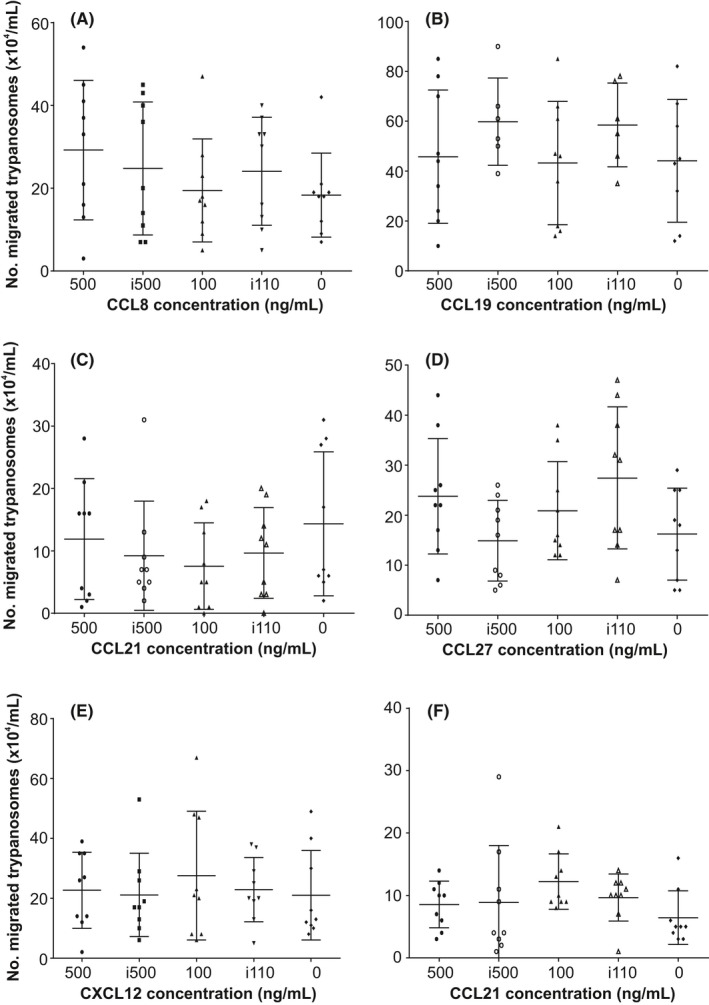
Effect of in vitro chemokine exposure on trypanosome chemotaxis. 1 × 10^6^ viable monomorphic *T brucei* Lister 427 trypanosomes (A‐E) or pleomorphic *T brucei* STIB 247 trypanosomes (F) were placed in the upper chamber of each well which was separated from the lower chamber by a 3 μm pore membrane. The respective concentration of (A) CCL8, (B) CCL19, (C,F) CCL21, (D) CCL27 and (E) CXCL12 were then added to the medium in the lower chamber and the number of trypanosomes which had migrated into the lower chamber determined 2 h later. Heat‐inactivated chemokine (i500, i100) or medium alone were used as negative controls. Each point represents the mean from triplicate wells, and the horizontal bar represents the mean ± SD. All experiments were repeated three times on different days

To determine whether certain chemokines may act as chemoattractants for *T brucei*, standard chemotaxis assays were performed. Monomorphic *T brucei* Lister 427 trypanosomes were placed in the upper chamber of each well, which was separated from the lower chamber by a 3 μm pore membrane. Differing concentrations of each chemokine were then added to the medium in the lower chamber and the number of trypanosomes that had migrated into the lower chamber was determined 2 hours later. Heat‐inactivated chemokines and medium alone were used as controls. The chemokines CCL8, CCL19, CCL21, CCL27 and CXCL12 did not stimulate significant chemoattraction of *T brucei* when compared to controls (Figure**** 3). This observation was not specific to the monomorphic *T* *brucei* Lister 427 parasite strain since a parallel set of experiments showed that CCL21 also exerted no significant chemoattraction towards the pleomorphic *T brucei* STIB 247 strain (Figure**** 3F). As anticipated, these chemokines mediated significant chemoattraction of mouse splenocytes (Figure S1).

**Figure 4:**
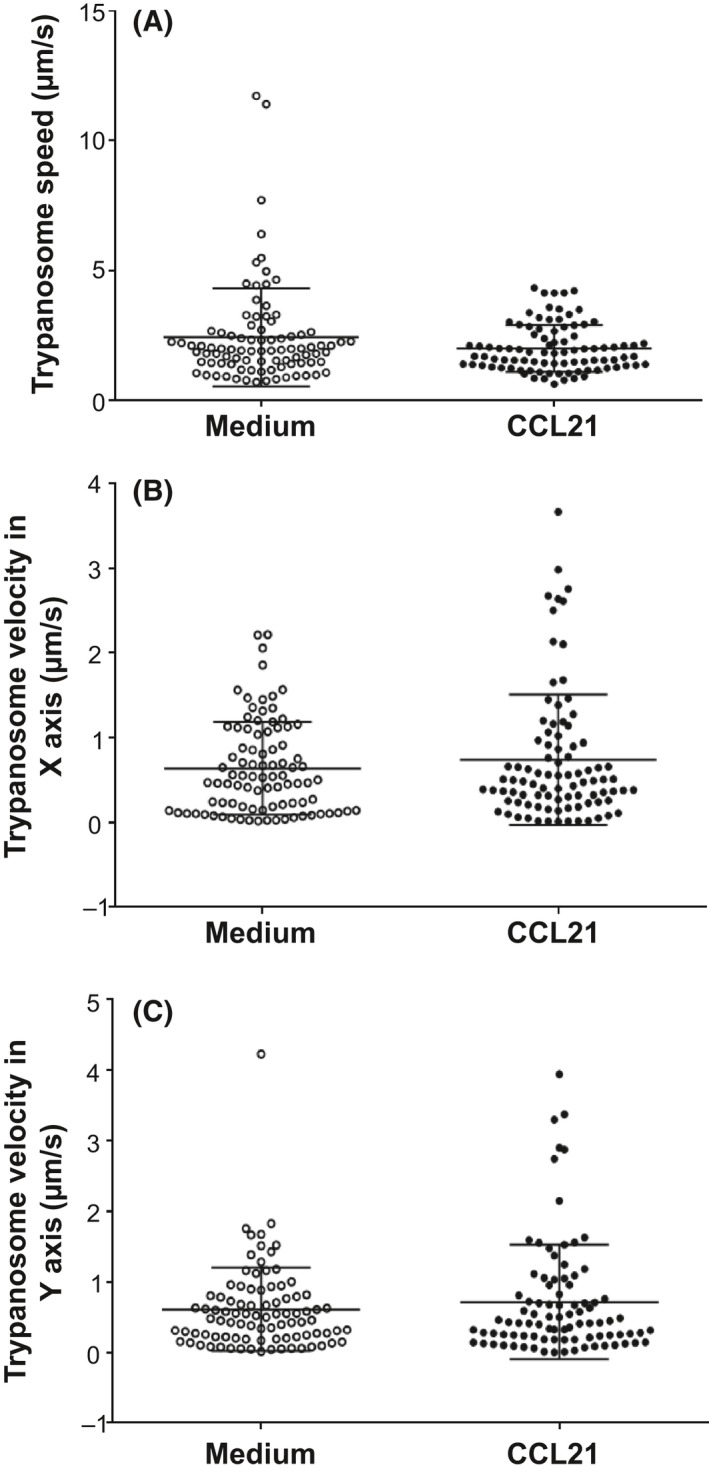
Effect of in vitro CCL21 exposure on trypanosome motility. Monomorphic *T brucei* Lister 427 trypanosomes were incubated in medium containing CCL21 for 2 h and the motility of 90 individual parasites recorded by live cell imaging. Imaris software was then used to determine the effects of treatment on trypanosome speed, as well as their velocities in the X and Y axes. Each point represents data from the analysis of individual trypanosomes, and the horizontal bar represents the mean ± SD

We also assessed whether exposure to these chemokines might alter trypanosome motility characteristics. The chemokine CCL21 was used in these studies due to its role in stimulating the homing of lymphocytes/leucocytes to lymphoid tissues and their migration across the vascular endothelium.^19^ Monomorphic *T brucei* Lister 427 trypanosomes were incubated in medium containing CCL21 for 2 hours, and the motility of 90 individual parasites was recorded by live cell imaging. Imaris software was then used to determine the effects of treatment on trypanosome speed, as well as their velocities in the X and Y axes. Consistent with data presented in Figure 3, our analysis clearly showed that CCL21 exposure did not significantly affect trypanosome speed or velocity when compared to control‐treated trypanosomes (Figure 4).

### 3.4 Effects of in vitro chemokine exposure on trypanosome viability

**Figure 5:**
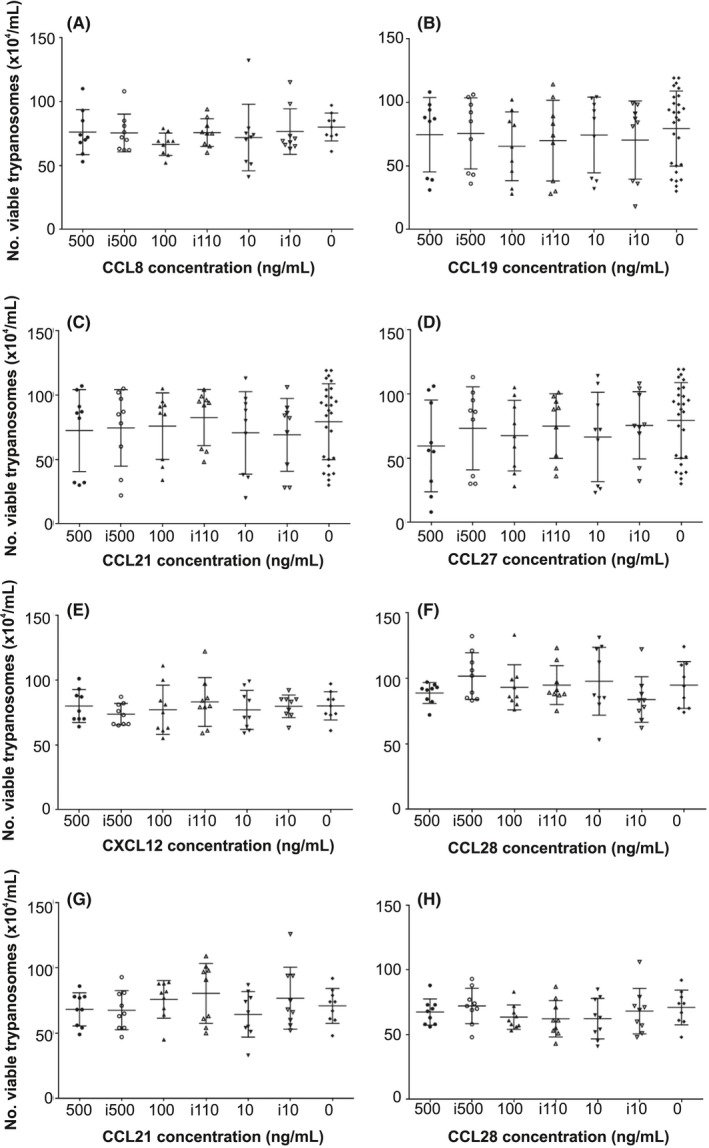
Effect of in vitro chemokine exposure on trypanosome viability. Viable monomorphic *T b brucei* Lister 427 trypanosomes (A‐F) or pleiomorphic *T brucei* STIB 247 trypanosomes (G,H) (8 × 10^5^/1 mL well) were incubated in medium containing differing concentrations of (A) CCL8, (B) CCL19, (C,G) CCL21, (D) CCL27, (E) CXCL12 and (F,H) CCL28 were then added to the medium and the number of viable trypanosomes determined 2 h later. Heat‐inactivated chemokine (i500, i100, i10) or medium alone were used as control. Each point represents the mean from triplicate wells, and the horizontal bar represents the mean ± SD. All experiments were repeated three times on different days

As well as mediating the migration and position of certain host cell populations within tissues, some chemokines can display antimicrobial activities towards a range of microbial pathogens.^12^, ^15^, ^20^, ^21^ We therefore determined whether chemokines might also exhibit direct parasiticidal effects. Monomorphic *T brucei* Lister 427 trypanosomes were incubated with differing concentrations of each chemokine and the number of viable trypanosomes determined 2 hours later. In these experiments, the chemokine CCL28 was also included since it had been shown in an independent study to exert potent antimicrobial effects towards bacteria and fungi^21^, ^22^ and the related protozoan parasite *L mexicana*.^15^ Heat‐inactivated chemokines and medium alone were used as controls. Our data show that the viability of *T brucei* was not significantly affected after in vitro exposure to CCL8, CCL19, CCL21, CCL27, CCL28 and CXCL12 (Figure**** 5). As above, this was not specific to the monomorphic *T brucei* Lister 427 strain of trypanosomes as CCL21 and CCL28 also exerted no significant effect on the pleomorphic *T brucei* STIB 247 strain (Figure**** 5G,H).

### 3.5 Effects of in vitro chemokine exposure on membrane permeability and integrity

**Figure 6:**
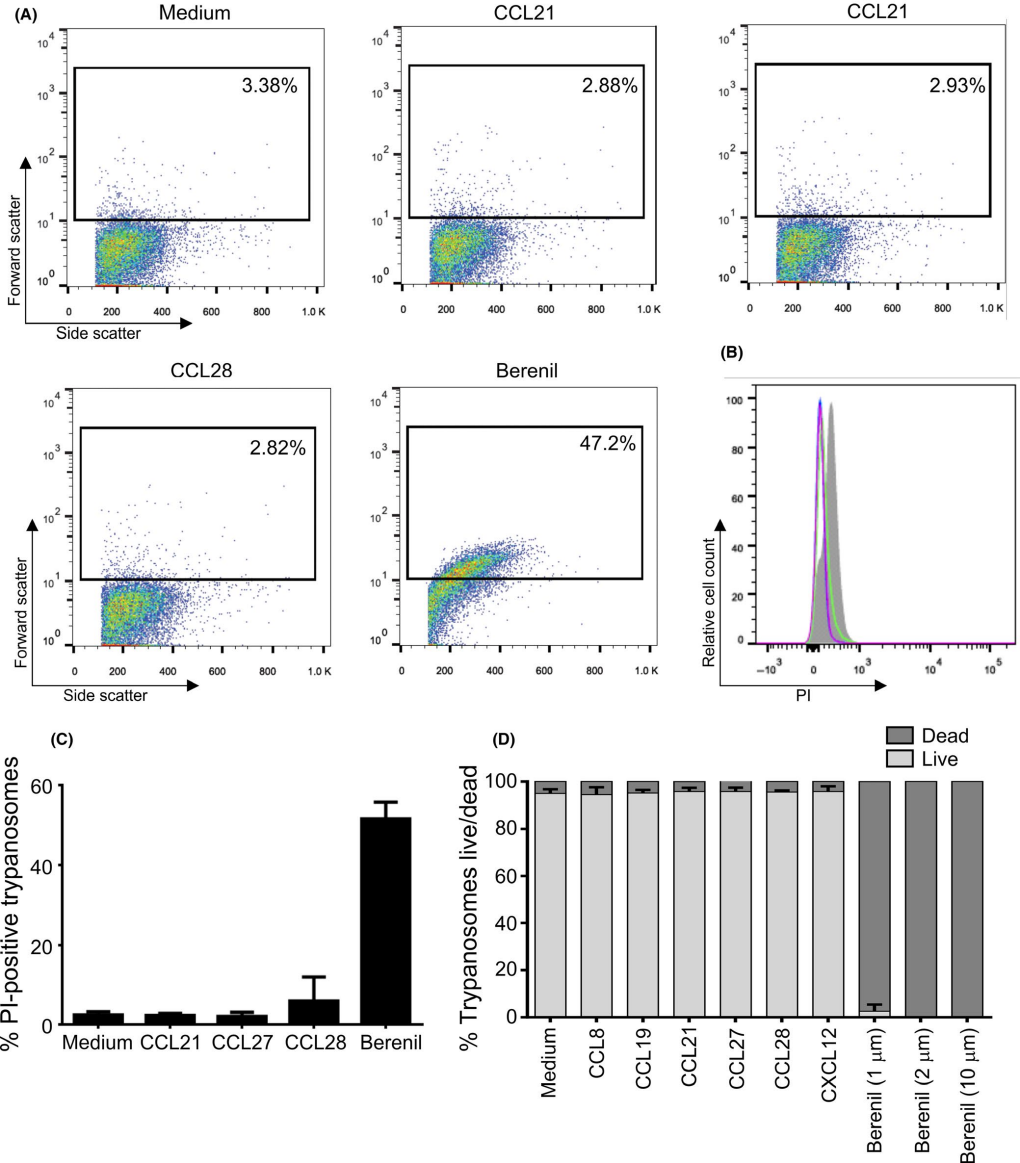
Effect of in vitro chemokine exposure on trypanosome membrane permeability and viability. A, Flow cytometric analysis of PI uptake by trypanosomes after chemokine exposure. *T brucei* Lister 427 trypanosomes were exposed for 2 h to 500 ng/mL of CCL21, CCL27 or CCL28, or 10 μmol/L of the anti‐trypanosome drug Berenil (diminazene aceturate) before analysis. The percentage PI positive cells in the gated regions of each scatter plot are shown. B, Histoplot shows the relative cell count vs PI uptake following exposure of *T brucei* Lister 427 trypanosomes to medium alone (control, red), CCL21 (light blue), CCL27 (dark blue), CCL28 (green) or Berenil (shaded). C, Histogram shows the % PI‐positive trypanosomes following in vitro exposure to chemokines or the trypanocidal drug Berenil. Each bar represents the mean from three independent experiments ± SD. D, Histogram shows the percentage live and dead trypanosomes after exposure to chemokines or the anti‐trypanosome drug Berenil. All experiments were repeated three times on different days

Some chemokines have been shown to mediate their antimicrobial effects by causing direct damage to the plasma membranes of the target microorganism, including the protozoan parasite *L mexicana*.^15^ We therefore determined whether the membrane integrity of chemokine‐treated *T brucei* parasites was affected by assessing their uptake of the vital dyes propidium iodide (PI) and trypan blue. Flow cytometry was used to determine the number of trypanosomes which were sufficiently permeabilized after treatment to allow entry of PI (Figure 6A‐C), whereas trypan blue‐exclusion was used to compare the effects of treatment on the number of live or dead trypanosomes (Figure**** 6D). Trypanosomes exposed to medium alone were used as controls. When trypanosomes were treated with the trypanocidal drug Berenil (diminazine aceturate, as a positive control) membrane integrity, and trypanosome viability was dramatically affected as anticipated (Figure**** 6). However, exposure to the chemokines CCL8, CCL19, CCL21, CCL27, CCL28 and CXCL12 had no significant effect on membrane integrity when compared to control‐treated trypanosomes (Figure**** 6).

**Figure 7:**
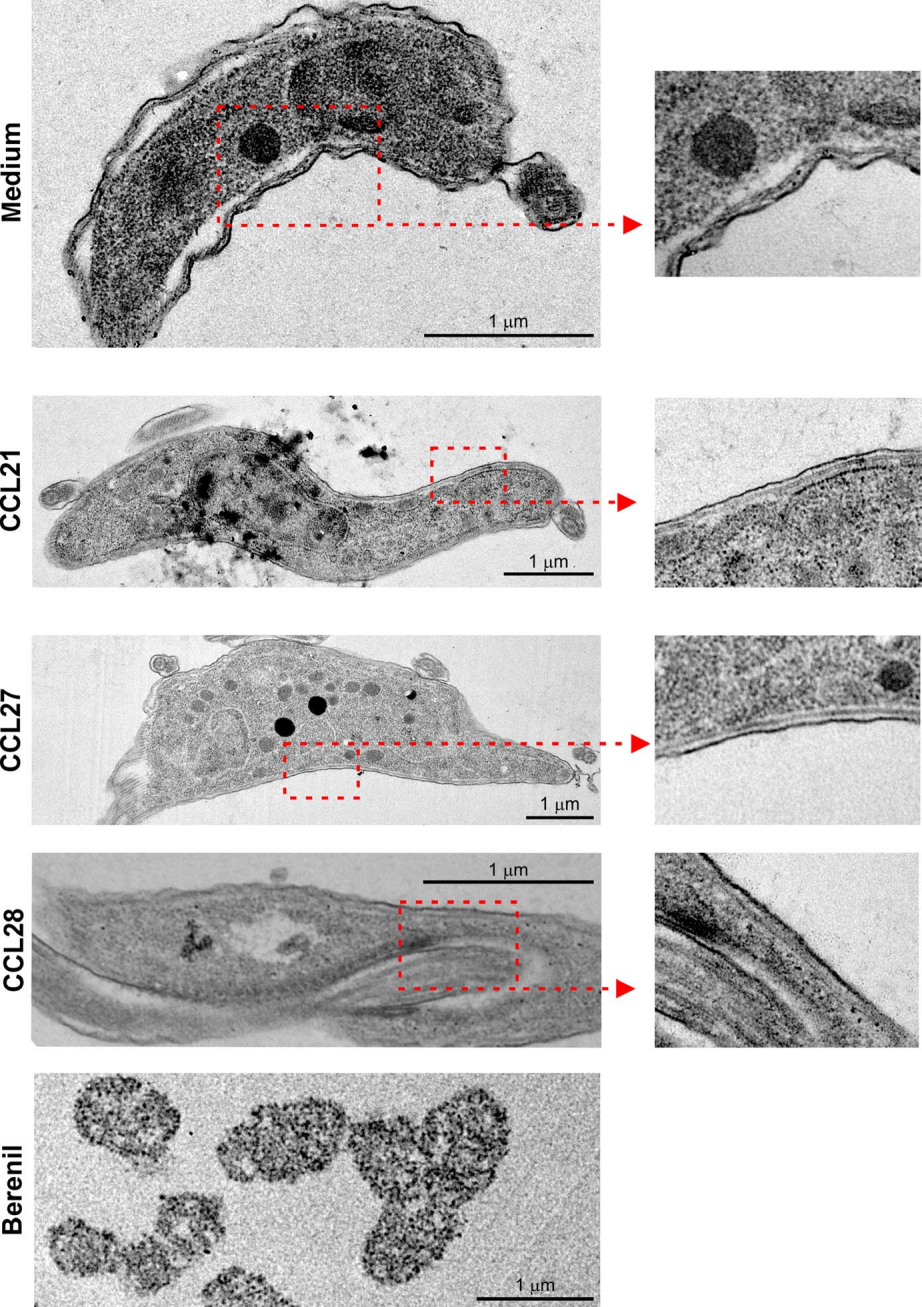
TEM analysis shows in vitro chemokine exposure does not adversely affect trypanosome morphology. *T brucei* Lister 427 trypanosomes were exposed to medium alone (control), or CCL21, CCL27 or CCL28 (500 ng/mL), or the anti‐trypanosome drug Berenil (diminazene aceturate, 10 μmol/L) for 2 h before analysis. Whereas substantial parasite destruction was observed following Berenil treatment, no observable effects on membrane integrity were observed after chemokine treatment

Finally, TEM was used to determine whether chemokine exposure caused morphological damage to the plasma membranes of the trypanosomes. As anticipated, substantial trypanosome destruction was observed following in vitro treatment with the trypanocidal drug Berenil (Figure**** 7). However, no observable effects on membrane integrity were observed following treatment with the chemokines CCL21, CCL27 or CCL28 when compared to control‐treated trypanosomes (Figure**** 7).

## 4 DISCUSSION

Understanding the nature of and cues that trigger trypanosome movement in and between different tissue compartments is poorly understood, despite being critical to the progression of the parasite's life cycle in the mammalian and insect hosts. This aspect of trypanosome behaviour is of particular interest given recent studies that have described tissue‐specific populations (skin and adipose),^2^, ^23^, ^24^ highlighting gaps in our knowledge of where and how trypanosomes disseminate in the mammalian host. Data in the current study show that following injection into the skin many of the trypanosomes could be observed migrating directly towards or away from the lymphatic vessels. Furthermore, some of the trypanosomes were also detected within the afferent lymphatic vessels in the dermis. These data are consistent with those in an independent study which has shown that following the intradermal injection of *T brucei* parasites by tsetse fly bite, the parasites were first detected within the draining lymph nodes within hours of infection before their subsequent detection within the bloodstream.^2^ Leucocytes and lymphocytes are specifically attracted to lymphatic endothelial cells along chemokine gradients, and their interactions with specific adhesion molecules coordinate their adhesion to and migration across the endothelium.^25^, ^26^ Given that some trypanosomes were observed migrating within the lymphatics we hypothesised that the parasites may be responsive to host chemokines and use them to aid their dissemination from the site of infection. However, our in vitro studies show that the chemokines CCL8, CCL19, CCL21, CCL27 and CXCL12 do not stimulate the chemotaxis or influence the motility of *T brucei*. This effect was evaluated using bloodstream forms of both monomorphic *T brucei* 427 and pleomorphic *T brucei* 247 parasite. Thus, our data suggest that the parasites are unlikely to use these chemokines as cues to aid their dissemination from the injection site in the skin.

Data from elegant in vitro high‐speed fluorescence microscopy studies have shown that the mode and dynamics of trypanosome locomotion can be influenced by the density of the surrounding matrix.^27^ In blood, the trypanosomes display efficient forward motion^27^ and this appears important to help avoid immune‐mediated clearance.^28^ However, when the parasites become trapped, such as in a densely packed environment that resembles collagen networks or tissue spaces, the parasites reverse their flagellar beat and swim backwards.^27^ This apparent ability of the trypanosomes to adjust the beating direction of their flagellum in response to mechanical cues may help to explain the motility characteristics of the parasites that we observed following their injection into the skin. Within the dermis, the trypanosomes could be visualised migrating towards and away from the lymphatics, with individual parasites repeatedly moving towards, then away, then back again. Furthermore, the trypanosomes within the lymphatics had significantly faster velocity when compared to those in the extra‐lymphatic environment.

A recent study showed that there was a transient upregulation of the genes encoding CXCL1 and CXCL5 in the dermis of mice after bites from *T brucei* infected tsetse flies.^29^ We did not test the effects of these chemokines on trypanosome motility or viability in the current study. However, since these chemokines are typically produced by epithelial cells in response to damage to recruit cells such as neutrophils,^30^ we consider they would be unlikely to contribute to the specific chemoattraction of trypanosomes towards lymphatic vessels.

Infection in the mammalian host is initiated by the intradermal injection of metacyclic trypomastigotes by the tsetse fly vector. While the precise timing is uncertain, these parasites then undergo morphological change into the long slender bloodstream forms that are adapted for survival within the mammalian host. The chemotaxis studies utilized in vitro cultivated bloodstream trypanosome forms, thus it is plausible that the chemokines tested may differ in their activity against metacyclic forms. However, an in silico protein‐protein sequence comparison (NCBI BLAST search) found no matches for homologues of murine chemokine receptors in *T brucei* genome data (data not shown).

The endothelium of the collecting initial lymphatics typically has incomplete or absent intercellular junctions. The loosely connected, overlapping borders of these lymphatic endothelial cells are considered to facilitate the unidirectional entry of tissue fluid and proteins into the lymphatics along hydrostatic pressure and protein gradients.^31^, ^32^ While it is plausible that African trypanosomes migrate towards the lymphatics by sensing lymph flow, this current is insufficient to push leucocytes such as classical dendritic cells towards the initial lymphatics.^26^

*Batrachochytrium dendrobatidis* is an important fungal pathogen of amphibians. The zoospores of this pathogen have been shown to exhibit chemotaxis towards nutritional cues including sugars, proteins and amino acids.^33^ Within the mammalian host, *T brucei* derives its metabolic energy from blood glucose using a unique form of glycolysis.^34^ Since the concentration of glucose in the lymph has been shown to be higher than the bloodstream,^35^ it is possible that glucose may act as a molecular cue for *T brucei* following injection into the skin.

Increasing evidence shows that many chemokines also possess potent antimicrobial properties against certain pathogens.^10^ All of the chemokines tested for parasiticidal activity in this study have been shown to demonstrate this activity: CCL8 and CCL19 are bactericidal against the Gram‐negative bacterium *E coli* 11; CCL21 is bactericidal against *E coli* and the Gram‐positive bacterium *S aureus* 11; CCL27 has fungicidal activity against *Candida albicans* 22; and CXCL12 is bactericidal against *S aureus* and the *E coli*.^11^ The chemokine CCL28 was of particular interest since it has been shown to exert broad‐spectrum antimicrobial effects towards Gram‐positive bacteria and Gram‐negative bacteria,^21^, ^22^ fungicidal activity towards *C albicans* 22 and parasiticidal activity towards the protozoan parasite *L mexicana*.^15^ However, none of the chemokines tested in the current study exerted any observable parasiticidal effects towards *T brucei*. Many of these antimicrobial chemokines mediate their activities through the disruption and lysis of the pathogen cell membrane.^10^ However, in our studies the membrane integrity of *T brucei* was not adversely affected after chemokine exposure. The human chemokines CXCL2, CXCL6, CXCL9, CXCL10, CCL20 and CCL28 have been shown to exert their parasiticidal effects against *L mexicana* by adversely affecting mitochondrial activity.^15^ However, a similar mode of action against bloodstream *T brucei* is unlikely since this life cycle stage lacks significant mitochondrial function.^36^ Although the above *Leishmania* study investigated the effects of chemokines on the promastigote forms of parasite which share similar dimensions, flagellar motility and extracellular niche to that of *T brucei*, it is plausible that antimicrobial chemokines may be more effective against intracellular pathogens. Within cells, the intracellular chemokine concentrations may be much higher, or the chemokine may disturb the intracellular niches in which the pathogens reside. For example, human CXCL4 can kill erythrocyte‐inhabiting *Plasmodium falciparum* parasites by selectively lysing the parasitic digestive vacuole.^37^

Together, our data show that following injection into the skin some of the trypanosomes could be observed migrating intravascularly within the afferent lymphatic vessels in the dermis. However, the cues that the trypanosomes might exploit to enable them to infect the lymphatic vessels are not known. Identification of the mechanisms used by African trypanosomes to establish host infection after intradermal injection into the skin would aid the development of novel prophylactic approaches to block their systemic dissemination in the mammalian host and reduce disease transmission.

## DISCLOSURES

None.

## AUTHOR CONTRIBUTIONS

NAM, LJM, PG and JMB conceived and designed the study; OAA and OA performed the study; OAA, TP, JB, LJM and NAM analysed and interpreted the data sets; all authors contributed to the writing of the manuscript; all authors approved the final version of the manuscript.
